# Prescription Patterns in Adolescent Patients With Depressive Disorder at a Tertiary Care Centre in Singapore

**DOI:** 10.1192/bjo.2024.699

**Published:** 2024-08-01

**Authors:** Tji Tjian Chee, Mark Ye Han Ngoh, Yew Ron Hoe, Crystal Xue Qi Chong, Pei Sheuan Vanessa Tan

**Affiliations:** National University Hospital, Singapore, Singapore

## Abstract

**Aims:**

We aim to examine the prescription patterns of local psychiatrists for adolescent patients with depressive disorder, treated on an outpatient basis, as part of an integrated programme for management of mood disorders in adolescent patients (IPMDA) in Singapore.

**Methods:**

An longitudinal cohort observational study was carried out at a psychiatry out-patient department in the National University Hospital of Singapore, as part of a specialised programme for the treatment of adolescents, aged 13 to 18, with depressive disorder. The data which were collected included information about age, gender, education, therapy provision and drug prescription included generic name and dosing.

**Results:**

A total of 129 patients were included in the study. 81.7% (n = 105) were females and 18.3% (n = 24) were males. 72 (76.4% female, 23.6% male) patients were started on medications. All patients were initiated on a monotherapy regime of antidepressants.

The most commonly prescribed antidepressant was Fluoxetine (58.3%), followed by Sertraline (18.1%), Fluvoxamine (12.5%), Escitalopram (6.9%), Mirtazapine (2.8%) and Amitriptyline (1.4%).

**Conclusion:**

Our findings revealed that current psychopharmacology practice for depressive disorder in Singapore generally follows the published Singaporean treatment guidelines, which is generally kept up to date with wider international recommendations.

The factor of pricing may affect the lower prescription of certain medications, such as Escitalopram, as it is more expensive than the other prescribed medications in the list.

